# Not just a colourful metaphor: modelling the landscape of cellular development using Hopfield networks

**DOI:** 10.1038/npjsba.2016.1

**Published:** 2016-02-18

**Authors:** Atefeh Taherian Fard, Sriganesh Srihari, Jessica C Mar, Mark A Ragan

**Affiliations:** 1 Institute for Molecular Bioscience and ARC Centre of Excellence in Bioinformatics, The University of Queensland, St Lucia, Brisbane, QLD, Australia; 2 Department of Systems & Computational Biology, Albert Einstein College of Medicine, Bronx, NY, USA; 3 Department of Epidemiology & Population Health, Albert Einstein College of Medicine, Bronx, NY, USA

## Abstract

The epigenetic landscape was introduced by Conrad Waddington as a metaphor of cellular development. Like a ball rolling down a hillside is channelled through a succession of valleys until it reaches the bottom, cells follow specific trajectories from a pluripotent state to a committed state. Transcription factors (TFs) interacting as a network (the gene regulatory network (GRN)) orchestrate this developmental process within each cell. Here, we quantitatively model the epigenetic landscape using a kind of artificial neural network called the Hopfield network (HN). An HN is composed of nodes (genes/TFs) and weighted undirected edges, resulting in a weight matrix (*W*) that stores interactions among the nodes over the entire network. We used gene co-expression to compute the edge weights. Through *W*, we then associate an energy score (*E*) to each input pattern (pattern of co-expression for a specific developmental stage) such that each pattern has a specific *E.* We propose that, based on the co-expression values stored in *W*, HN associates lower *E* values to stable phenotypic states and higher *E* to transient states. We validate our model using time course gene-expression data sets representing stages of development across 12 biological processes including differentiation of human embryonic stem cells into specialized cells, differentiation of THP1 monocytes to macrophages during immune response and trans-differentiation of epithelial to mesenchymal cells in cancer. We observe that transient states have higher energy than the stable phenotypic states, yielding an arc-shaped trajectory. This relationship was confirmed by perturbation analysis. HNs offer an attractive framework for quantitative modelling of cell differentiation (as a landscape) from empirical data. Using HNs, we identify genes and TFs that drive cell-fate transitions, and gain insight into the global dynamics of GRNs.

## Introduction

In the course of development, cells take on a succession of distinct phenotypic states, from an initial totipotent or pluripotent state through to a final differentiated state in which the cell is committed to a particular location and function. This commitment is typically progressive, with cells passing through a hierarchy of increasingly specialized intermediate states along a developmental trajectory. The transition from one intermediate state to the next is driven by the concerted action of transcription factors (TFs) and other biomolecules as part of the gene regulatory network (GRN).

Conrad Waddington introduced the epigenetic landscape as a metaphor for cellular development.^1^ Like a population of balls rolling down a rough hillside, cells follow specific trajectories (valleys) and encounter decision points (inflections) before eventually coming to rest in one or another potentially steady state, termed attractors.^2,3^ Importantly, Waddington depicted the topography of the landscape as determined by a system of underpinning interconnected cables. Although this metaphor preceded our current understanding of the relationship among genes, transcripts and proteins, it is easily interpretable today as depicting the framework for control of cellular differentiation and phenotype by dynamics of the underlying GRN.

To model this landscape, Huang^4^ proposed a “quasi-potential” that connects the elevation of the landscape to the likelihood of the corresponding cell state. For Huang^4^, each point in the landscape represents a gene-expression configuration of a binary regulatory circuit. An alternative formulation^5^ emphasises the possibility of cell trajectories without necessarily “rolling downhill”, e.g., the landscape is modelled as a non-hierarchical “epigenetic disc” in which cell fates can be interconverted without necessarily traversing back up through a developmental hierarchy. Other formulations emphasise the underlying molecular mechanisms including the role of DNA methylation, histone modification and signalling pathways in driving cell-fate decisions.^6–8^

Modern experimental evidence does in fact show hierarchies of cell fates^9^ with TFs driving cell development. For instance, Takahashi and Yamanaka^10^ demonstrated that small sets of TFs (the “Yamanaka cocktail”) are sufficient to induce pluripotency in a differentiated somatic cell, i.e., the cell is reprogrammed back to its original state at the top of Waddington’s landscape. Experimental protocols exist for generating specialized cells including neurons,^11^ hepatocytes,^12^ macrophages^13^ and cardiomyocytes from undifferentiated fibroblasts^14^ using small sets of TFs, demonstrating cell-fate conversion and reprogramming.

Nonetheless it remains controversial whether a Waddington (or similar) landscape can be computed solely from empirical data. The view of Waddington’s landscape as a “colourful metaphor”^15^ that cannot be quantified has been echoed by several authors.^15–17^ The recent availability of single-cell transcriptomic (and other omic) time course data provides new opportunity to explore landscape models that could provide insight into the GRNs that underpin cellular development. However, there are issues in quantifying this landscape, including design of the algorithmic framework *per se*, the approach to data utilisation and mapping of actual developmental stages to features (e.g., attractors) of landscape models.

Dynamic systems of biomolecular interactions have been modelled using Bayesian networks,^18–20^ generalised logical networks,^21^ constraint-based models,^22^ Petri nets,^23^ stochastic master equations^24^ or agent-based approaches.^25^ Although these approaches can identify stable states, in general they do not generate a solution landscape of the sort introduced above.

In the context of GRNs and cell fate, attractor landscapes have been described using Boolean networks,^26–28^ neural networks^29–31^ or systems of ordinary differential equations (ODEs);^32^ however, these have seen application mostly in computational simulation rather than in the analysis of empirical data. For instance, Wang *et al.*^33^ used ODEs and simulated data to construct a probabilistic “pseudo-potential” energy landscape using only two TFs, GATA1 and PU.1, to identify the developmental path of cells from undifferentiated to differentiated states. This system permits a binary cell-fate decision such as macrophage/monocyte or megakaryocyte/erythrocyte. Similar probabilistic potential landscapes have been used to model lysis-lysogen switching in bacteriophage λ^34^ and the mitogen-activated protein kinase (MAPK) signal transduction network.^35^ Srihari *et al.*^36^ used binarized gene-expression data in an optimisation framework to derive attractors of cell states and identify TFs switched between these attractors. Ferrell^37^ proposed an alternative landscape for the developmental processes of cell-fate induction and inhibition, in which cell-fate commitment corresponds to the disappearance of a valley rather than the creation of a new valley. Davila-Velderrain *et al*.^38^ provide a comprehensive overview. Problems associated with these models include (lack of) computational scalability and, for ODEs, the need for rate constants.

Mathematical models and GRNs have their own descriptions of states, trajectories and attractors. Questions pertain to whether and how phenotypic states of a cell, which are maintained and regulated by a GRN, can be mapped to an attractor landscape model computed from gene-expression and/or other empirical data. Here we use Hopfield networks (HNs) to model an attractor landscape that can serve as a framework to understand the dynamics of the underpinning GRN.

HNs, introduced by John Hopfield in 1982, are auto-associative (recurrent) artificial neural networks. Input patterns to the HN are associated with distinct attractors of the network, and can later be recalled even from partial or noisy inputs. Koulakov and Lazebnik^29^ used an HN to simulate fusions between different types of cells corresponding to distinct attractors of the HN, concluding that fusion helps the cells reach an attractor state that would otherwise be inaccessible. Lang *et al.*^31^ used a similar approach to model and explain partially reprogrammed cell fates, and identified driver TFs involved in this process. They employed binarized gene-expression values, based on a conditional probability distribution derived from global histone-modification data, which reflect the epigenetic state of TFs and developmental signals in the landscape. Maetschke and Ragan,^30^ on the other hand, constructed an HN from static gene-expression data for different subtypes of cancer, and showed that cancer subtypes can be characterized as distinct attractors of an HN.

Here we construct HNs from large-scale gene-expression time course data, and map developmental trajectories to Hopfield energy profiles in this landscape. We find that after cells are induced to differentiate, parts of their GRNs become less tightly correlated, as expected for a cell in transition from one cell-fate to another. The topography of these landscapes reflects the correlated activity of key genes that have been experimentally shown to drive cell-fate transitions and decision-making.

## Results

Here we describe the HN energy landscape generated from the first case study.

### Differentiation of HES2 stem cells

**Figure 1:**
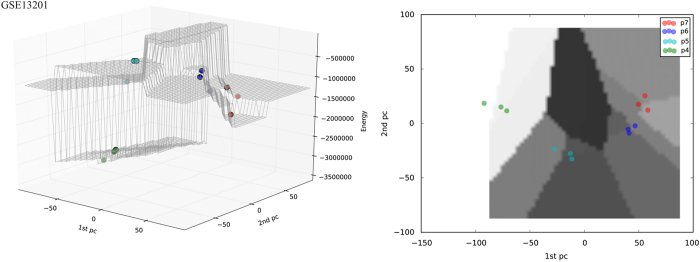
Hopfield energy landscape of the first case study (GSE13201) from two different perspectives. The *x* and *y* axes represent the first and second principal components of the data; the *z* axis represents the energy *E*. The left panel show side views of the landscape; the elevation of each group on the landscape is clearly visible. The right panel shows the top view of the landscape; the trajectory of cell movement is visible in the (*x*,*y*) plane. P7 (*E*_P7_=−1320897) cells are positioned at the lowest level of the Hopfield energy landscape. The higher energies of the transient groups (P6 and P5, *E*_P6_=−755220 and *E*_P5_=−599724) are followed by a decrease in energy to P4 (*E*_P4_=−3307223) resulting in the observed trajectory.

**Figure 2:**
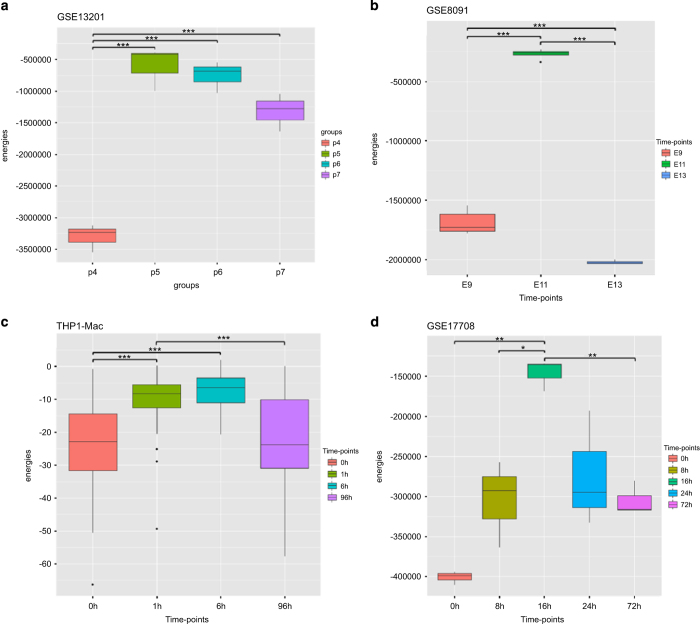
Differences between the mean of energy values for cell stages in the four case studies. (**a**, GSE13201; **b**, GSE8091; **c**, THP1-Mac and **d**, GSE17708. Significance: *p*-value=0 “***”, *p*-value<0.001 “**”, *p*-value<0.01 “*”).

This data set consisted of 12 samples in 4 groups, assayed with 48,687 expression probes of which 3,753 remained after feature selection. Figure 1 shows the energy landscape of fractionated HES2 stem cells. For cells of group P7, which are dormant but inducible to a pluripotent state, we observe a relatively low-energy score *E*_P7_=−1320897, i.e., a low elevation on the Hopfield energy landscape. On induction, these cells enter intermediate state P6 followed by P5, where we see an increase in energy to *E*_P6_=−755220 and *E*_P5_=−599724 before the cells reach the differentiated state P4 with the lowest energy *E*_P4_=−3307223 (Figure 2a). This progression traces a trajectory on the Hopfield energy landscape. Two views of this trajectory are shown in Figure 1: the left plot shows the three-dimensional view of the Hopfield energy landscape, while the right plot shows the (inferred) trajectory driven by changes in expression of the genes that contribute to principal component (PC) 1 and 2.

**Figure 3:**
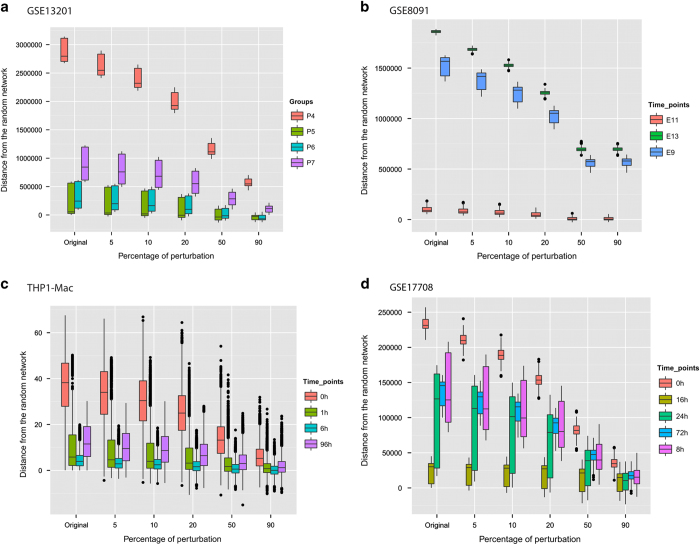
Perturbation analysis of time-specific networks for each data set. **a**, GSE13201; **b**, GSE8091; **c**, THP1-Mac and **d**, GSE17708. The *y* axis represents the mean distance of the perturbed networks (100-fold) from the random network. We progressively randomized a subset of 5, 10, 20, 50 and 90% gene-expression values of randomly selected genes, and compared the energy values of the original networks to those obtained from randomly generated networks.

To ensure that the Hopfield energies indeed correspond to stages of differentiation as captured by the expression profiles (i.e., that low Hopfield energies represent stable phenotypic states and high energies transition states) and are not merely an artefact, we progressively perturbed the gene-expression values for each stage, and computed the energies of the resulting HNs (Figure 3a). If random perturbation does not alter the Hopfield energy significantly, that cellular phenotype is stable and the underlying network maps to an attractor in the Hopfield landscape. On the other hand, if the perturbed networks tend to have significantly different energies, or conversely if the energy of the network before perturbation is similar to that of the randomly perturbed states, then the cells are in a transient state that can be represented as a hill in the Hopfield landscape.

We observed that when 5% of the gene-expression values are perturbed, energies for the first (P7) and last (P4) groups show greater difference *vis-a-vis* the random network (Figure 3a), and maintain lower energy values. By contrast, energies of the transient groups P5 and P6 are significantly (each *P* value 0.0) closer to that of the random network at all investigated levels of perturbation up to 50% (Figure 3a). This demonstrates that attractors are robust to perturbation, and confirms that Hopfield energy profiles can describe profiles of cellular differentiation.

We identified TFs and genes that are potentially major drivers of cell-state transition by comparing their discretized expression values between successive stages, and checking to see if they have switched (from −1 to +1 or vice versa). The feature-selected genes that switch activity between groups at each transition are listed in Supplementary Table S2. Different numbers of genes are switched at each transition, reflecting the dynamic behaviour of the GRN during development.^39^ Table 1 lists the Gene Ontology (GO) Biological Process terms^40^ and KEGG pathways^41^ most over-represented among the top 100 genes switched between the first and last groups. Among the enriched GO terms are *cell-fate determination* and *cellular differentiation*.^42,43^ The KEGG pathways^41^ most-enriched were *Hedgehog signalling*, which governs a wide range of processes during embryonic development and controls stem-cell proliferation^44^ and *Wnt signalling*, a major player in stem-cell self-renewal and differentiation.^45^ Among the switched genes we find FOXA1, GATA4, CD9 and OCT4 TFs involved in *regulation of stem-cell differentiation* (DAVID^46,47^). Specifically, the differentiation markers FOXA1 and GATA4 are upregulated in P4, whereas the pluripotency markers CD9 and OCT4 are upregulated in P7 (Supplementary Figure S1).

**Figure 4:**
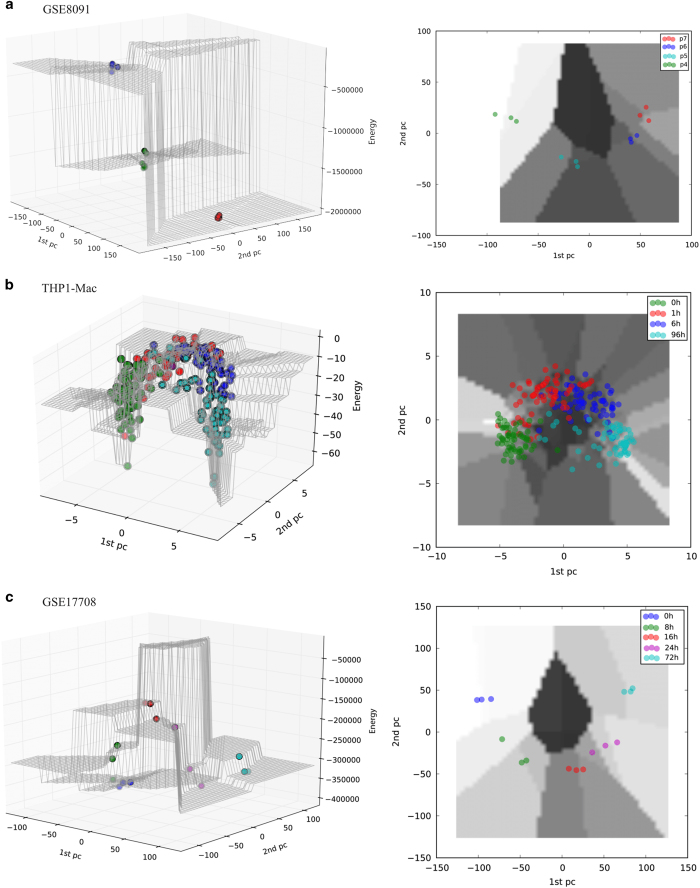
Hopfield energy landscape of **a**, the second (GSE8091), **b**, third (THP1-Mac) and **c**, fourth (GSE17708) case studies. The *x* and *y* axes represent the first and the second principal components of the data; the *z* axis represents the energy *E*.

For the remaining case studies, we inferred different genes to have switched expression, and different pathways and biological processes to be enriched, but we always observed the same pattern of energy changes across time points (Supplementary Tables S2 and S3). Results of the three other main case studies are presented in Figures 2,3,4 and Supplementary Figure S1, and Table 1 and Supplementary Table S2; for all others please see the Supplementary Material (Supplementary Tables S1, S3 and S4; Supplementary Figures S2 and S3).

## Discussion and Conclusion

We computed developmental landscapes from single-cell gene-expression data in 12 sets of time course data sets, using the mathematical formalism of the Hopfield recurrent neural network. Each point in each landscape represents the state of a cell; its elevation is determined by the energy *E*, which we compute from the pattern of co-variation of gene expression in that cell. For each landscape model, groups of cells at the same developmental stage, and thus sharing a common pattern of gene expression, are positioned close together. As we construct the landscapes based on the set of genes showing the greatest variation in expression across the time points in each data set, the models reflect the molecular biological processes underlying cell development.

In our HN models, the energy of a state space does not reflect its likelihood; instead, *E* measures the extent of co-variation over all pairs of feature-selected genes, capturing a distinct pattern of gene expression which represents a cellular phenotype.^48^ Strong co-variation yields a relatively low energy, whereas looser co-variation gives a high energy. Along a time course as the GRN is differentially regulated or rewired, changes in the patterns of co-variation are reflected as a trajectory on our landscape. For these 12 data sets, identities of the switched genes provide insights into functional modules, many of which (e.g., Hedgehog, Wnt and MAPK signalling pathways) have previously been validated experimentally.

We selected a form of perturbation analysis to build confidence in our energy values. The perturbation results make it clear that the energy values we compute for the initial and final states are far from those of random networks; this allows us to make comparisons among states. The set of energy values arising from perturbation analysis should not be taken as an estimate of the stability of an attractor, or the time or energy a cell needs to move out of that attractor state; for such concepts, new methods remain to be developed.

Different algorithmic frameworks are available to describe systems of biomolecular interactions. HNs are computationally simple, and our results demonstrate that they can capture trajectories of cellular development using only gene-expression data.

Following Maetschke and Ragan^30^ and Lang *et al.*^31^ we employed gene-expression data, typically at genome-wide scale, as input. Like these authors, we sought to reduce noise (and thereby avoid spurious attractors) and improve computability by first carrying out feature selection. Thus we based each analysis on the set of genes (probes) most-informative in its respective context. Lang *et al.*^31^ further used histone-modification data to establish a threshold for the binarization of gene-expression values. In principle, any type of data that provides patterns characteristic of cells at different developmental stages or time points could be input directly into the HN, including transcriptomic data for gene expression. Indeed it would be possible to use HNs to build a landscape model over mixed data types, yielding a unique approach to integrating data in developmental systems biology.

Models are abstractions of reality, and a mapping is required to link features of a model to events in a real process. Here, particular care is required to describe this mapping, as both the real physical GRN in cells, and the HN framework of our model, employ concepts of states, trajectories and attractors.^30^ At a given time, a cell is described by a state that reflects the overall pattern of interactions within its GRN. In the Hopfield framework, state refers to the value of *H*^(*t*)^(*s*), where *t* is the update cycle of the HN. Because our aim here is to position each cell on the landscape, we did not allow the HN to converge to its lowest energy, but instead use the observed expression pattern to compute *E*.

These data sets are not dynamic in the sense of measuring gene expression in a fixed set of cells through a succession of time points. Rather, we position snapshots of the system (Hopfield energy profiles) in a common landscape model, then draw on external information (e.g., sampling order from a population or expression of surface markers) to trace a temporal progression (trajectory) across the landscape.

In the HN formalism, attractors are local minima of the energy landscape, and in the present context correspond to phenotypic states maintained by the underlying GRN. Here, cells start out in a (perhaps artificially) stable state for which we calculate a low energy. After induction they progress through transient states described by higher energy values, and eventually reach another low-energy phenotype represented by an attractor. As in Waddington’s metaphor, in each data set we observed sets of cells positioned along a developmental trajectory, with each point on the landscape representing a state of the GRN at a specific time. While Waddington implied that the topography of his hillside might be dynamic (by depicting interconnected cables beneath it), here we employ a static weight matrix, so our cells (networks) map onto a static landscape. Moreover, unlike in the Waddington metaphor, the trajectories we infer do not run downhill into a globally low-energy attractor at one edge of a state-space dimension; rather, we find low-energy states at both ends of every trajectory.

In principle, the HN model can be applied to contexts other than normal cellular development. For example, following the study by Huang,^49^ who considered cancer as a pre-existing (but usually unvisited) attractor in his quasi-potential landscape, we might construct an HN from cancer-progression data and track trajectories of cells as they progress from a normal to a cancerous state. We could extend the model by employing targeted perturbation to measure the contribution of subsets of genes or TFs to the GRN, and thus to trajectories of disease progression.

As envisioned by Waddington, Kauffman and others, it is thus possible to compute a robust quantitative landscape model, based on empirical data, which reflects the collective behaviour of genes and TFs in driving cellular differentiation. By providing the framework for such models, HNs show that developmental landscapes need not be just a colourful metaphor.

## Materials and methods

### HN model: general background

An HN consists of nodes and weighted undirected interactions (represented as an interaction matrix) between these nodes. So-called patterns, for instance gene-expression profiles, can be stored as the weights of the network. Stored patterns (even when distorted) can then be retrieved from the network by a recurrent recall procedure.^50^ The similarity of an arbitrary input pattern to a stored pattern can be expressed by an energy function. Local minima of the energy function are the attractors of the system, to which input patterns converge during recall. A network can have multiple attractors with different minimum values. The HN associates similar stored patterns with the same attractor, whereas distinct stored patterns tend to be associated with different attractors.

Let *S* be a set of *m* samples (cells) under study, and let *G*={*g*_1_, *g*_2_,…,*g_n_*} be the set of genes profiled from these samples. For any sample *s*∈*S*, each gene is assigned to a node, thereby giving *n* nodes *H*(*s*)={*H*_1_, *H*_2_,_…_,*H_n_*}(*s*) in the HN. Each node *H*_*i*_ carries the expression value for *g*_*i*_ normalised and discretized to the values {−1, 0, 1}. For any pair of nodes (*H*_*i*_, *H*_*j*_), *H*_*i*_≠*H*_*j*_, we assign the interaction weight *w*(*H*_*i*_, *H*_*j*_) ∈ [−1,1] as the co-expression (computed here as Pearson's correlation) between {*g*_*i*_, *g*_*j*_} across the *m* samples and *w*(*H*_*i*_, *H*_*i*_)=0, resulting in a zero-diagonal symmetric weight matrix *W*. For each node *H*_*i*_, *N*(*H*_*i*_) is its set of neighbours (connected nodes).

Each sample *s*∈*S*, consisting of gene-expression values for the genes in *G*, can be stored by iterating through the HN. At each iteration, the node *H*_*i*_ is updated to $H_{i}=\sum_{j\in N(H_{i})}\left[H_{j}w(H_{i},H_{j})\right],$ the weighted sum across all connected nodes of *H*_*i*_. If *w*(*H*_*i*_, *H*_*j*_)>0 then *H*_*i*_ is updated to a value of the same sign (positive or negative) as *H*_*j*_, whereas if *w*(*H*_*i*_, *H*_*j*_)<0 then *H*_*i*_ is updated to a value of the opposite sign as *H*_*j*_. Consequently, *H*_*i*_ is either “pulled towards” or “pushed away” from *H*_*j*_ depending on *w*(*H*_*i*_, *H*_*j*_). After each update, we can capture the extent of agreement or disagreement between *H*_*i*_ and *H*_*j*_ as an energy function *E*(*H*_*i*_,*H*_*j*_)=−*H*_*i*_*W*_*ij*_*H*_*j*_, with the convention that lower values represent higher agreement or stability. Thus the energy for the entire network is given by $E\left[H(s)\right]=-1/2HWH^{T}$

Such an energy function belongs to the Lyapunov family of monotonically non-increasing functions,^51^ in which as the iterations progress and the values assigned to the nodes are repeatedly updated, *E*[*H*(*s*)] converges to a low-energy state. This converged energy value represents the attractor for the sample *s*, and *s* is said to have converged to its attractor. This energy value scales linearly and is unitless. Given this framework, if we have a collection of samples $S=\left\{S_{1},S_{2},\ldots ,S_{k}\right\}$ where each *S*_*i*_ represents a set of samples from a specific stage (or time point) of cellular differentiation, we expect all samples in *S*_*i*_ to converge to the same attractor in the HN.

### Hopfield model for cellular differentiation

Here, we aim to link the values of the HN energy function to stages of cellular differentiation. We hypothesise that as a cell transits from an initial to a differentiated state, the interplay (co-variation) between genes changes (in its GRN), and the cell as a network of genes moves along the Hopfield energy profile. By computing the energy values based on gene-expression profiles of samples at different stages of cellular development, we capture this transition. In particular, we demonstrate that if pair-wise co-expression coefficients are used to construct the weight matrix, these energy values indeed reflect the developmental stages of the cell. Tight correlation corresponds to lower energy values, and looser correlation to less-favourable energies.

Using sets of cells from different stages of differentiation (e.g., pluripotent to differentiated states), here we demonstrate that the computed energy level reflects the stage of differentiation. Our approach differs in two main respects from that presented in the study by Maetschke and Ragan,^30^ which was based on the standard Hopfield model: we build the weight matrix using Pearson's correlation rather than Hebbian learning, and omit the iteration step so as to compute energies that represent the actual biological states of the network rather than iterated values.

**Figure 5:**
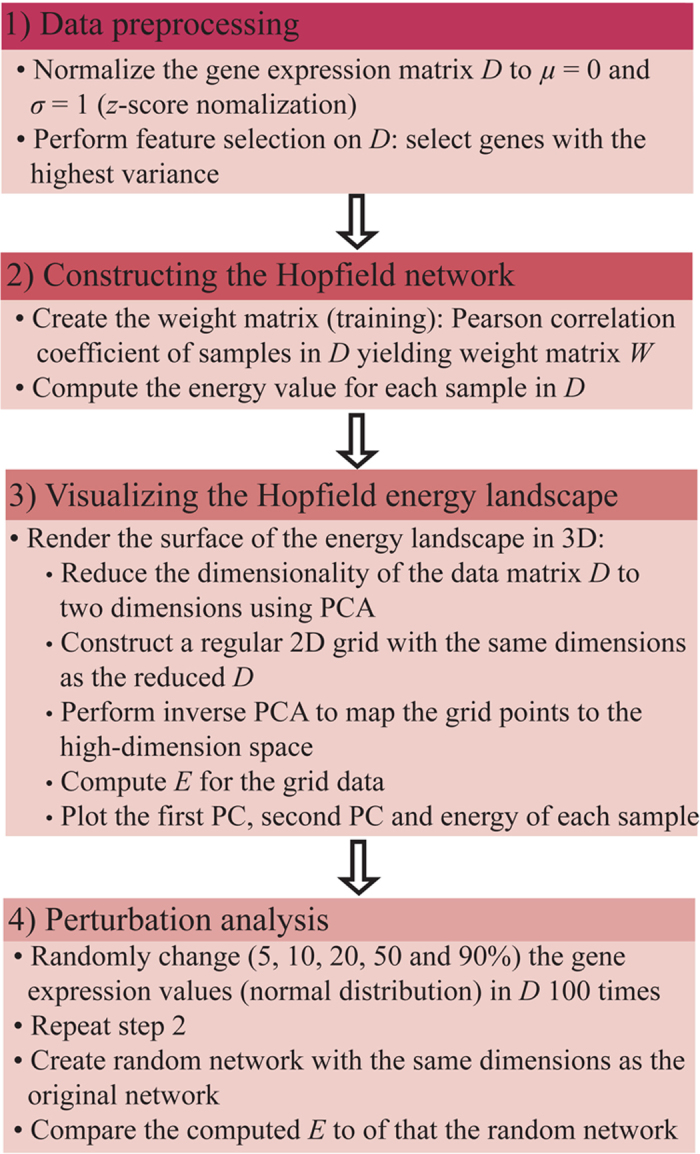
Workflow of the study. PCA, principal component analysis.

By visualising the transitions between these energy levels, we realise the landscape of cellular differentiation. The landscape is an *n*+1 dimensional space with *n* dimensions for the genes, and one dimension for the energy *E*(*H*). To visualise this landscape in three dimensions, we render the surface of the energy landscape by interpolating energy values over a two-dimensional surface: first the dimensionality of the gene-expression data is reduced to 2 using principal component analysis (PCA), a regular grid is constructed with the same dimensions as the reduced data, inverse PCA is performed to map the grid points to the high-dimensional space, then *E* is computed for the grid data.^30^ In this landscape, the two major principal components of the *n* genes serve as our dimensions *x* and *y*, and the energy is represented as the third dimension *z*. Stages *S*_*i*_ are points in this space, represented here by unique colours (Figure 5).

### Case studies and data sets

We constructed HNs for 12 time course data sets covering a broad range of case studies. Four of these (Table 2) are described here; for the others see Supplementary Material. The first case study^52^ analyses HES2 human embryonic stem (ES) cells. In this study, cells were fractionated by flow cytometry into different categories of pluripotency based on the expression of two stem-cell surface markers, GCTM-2 and CD9, yielding the four groups P7 (GCMT2^HIGH^-CD9^HIGH^), P6 (GCMT2^MID^-CD9^MID^), P5 (GCMT2^LOW^-CD9^LOW^) and P4 (GCMT2^−^-CD9^−^). Cells in group P7 are in a dormant state inducible to pluripotency, whereas P4 cells are committed to their lineage, and P6 and P5 cells are in intermediate states of differentiation. The data set includes expression profiles from 12 samples corresponding to 3 replicates from each category.

The three other case studies cover maturation of embryonic neural cells during mouse brain development, time course differentiation of THP1 monocyte cells to macrophages and trans-differentiation of epithelial to mesenchymal cells in cancer (Table 2). Please refer to the Supplementary Document for detailed description of these data sets. Eight additional case studies cover time course differentiation of mouse and human ES cells, induced pluripotent stem cells and organogenesis (Supplementary Table S1). For each data set, we performed *z*-score normalisation, followed by feature selection to extract the probes with the highest variation across groups and time points. To determine number of features we used the elbow of the variance plot over features, choosing the number such that including one more probe does not change the variance.

### Robustness analysis

For each data set, we assessed robustness of the energy values by randomly changing the expression values of a randomly selected subset (5, 10, 20, 50 or 90%) of the feature-selected genes. A new expression value was randomly chosen from within the interval {−1, +1}. We then constructed the HN network as above, and computed *E* for the network for each round of perturbation. We also compared the resulting energy value with that of a random network of the same size (Δ*E*=*E*_random_−*E*_perturbed_). This process was repeated 100 times for each data set and each proportion of perturbed genes. Please refer to the Supplementary Document for details.

## Figures and Tables

**Table 1:** Functional analysis of the switched genes across stages of differentiation/development

*Case study*	*#Selected probes*	*Transition*	*# Probes switched*	*Biological process*	P*-value*	*KEGG pathways*	P*-value*
GSE13201	3,753	P7–P4	3,405	Cell fate determination	7.72E−03	Hedgehog signalling pathway	1.3E−2
		P5–P4	1,861	Cellular differentiation	1.34E−09	Wnt signalling pathway	8.0E−2
		P6–P4	3,430	System development	9.96E−12	Basal cell carcinoma	1.3E−2
		P6–P5	2,712	Skeletal system development	1.77E−10		
		P7–P5	2,810	Central nervous system development	1.10E−09		
		P7–P6	1,453	Embryo development	9.54E−09		
GSE8091	2,748	E9–E13	2,594	System development	3.48E−06	Focal adhesion	8.1E−3
		E9–E11	2,169	Developmental process	1.06E−05		
		E11–E13	2,430	Cell differentiation	1.14E−04		
THP1-Mac	45	0–96 h	20	Cell differentiation	6.57E−13	Wnt signalling pathway	7.2E−5
		0–1 h	23	System development	1.05E−11	B-cell receptor signalling pathway	1.5E−3
		0–6 h	22	Haemopoiesis	4.88E−10	PPAR signalling pathway	1.9E−2
		1–6 h	23			MAPK signalling pathway	4.8E−2
		1–96 h	21				
		1–6 h	23				
		6–96 h	22				
GSE17708	2,620	0–72 h	1,974	Regulation of cell proliferation	1.82E−02	ECM-receptor interaction	7.1E−3
		0–8 h	1,511	Cell adhesion	1.80E−3		
		0–16 h	2,013	Developmental process	4.40E−01		
		0–24 h	2,021				
		8–16 h	1,806				
		16–24 h	1,606				
		16–72 h	1,737				
		8–24 h	1,936				
		24–72 h	1,478				
		8–72 h	1,960				

**Table 2:** Data sets for the four main case studies

*Study*	*data set*	*Summary*	*Microarray platform*	*# Of samples*	*Stages of differentiation/development*	*# Of probes*
Kolle *et al.*^52^	GSE13201	HES2 embryonic stem cells were sorted by the presence of GCTM-2 and CD9 into four populations using Fluorescence Activated Cell Sorting (FACS) method	Illumina Human-6 v2.0 Expression BeadChip (Illumina Inc., San Diego, CA, USA)	12	P7 (×3)	48,687
					P6 (×3)	
					P5 (×3)	
					P4 (×3)	
Hartl *et al.*^43^	GSE8091	Transcriptome of embryonic mouse brain development at 9.5, 11.5 and 13.5 embryonic days	Affymetrix Mouse Genome 430 2.0 Array (Affymetrix Inc., Santa Clara, CA, USA)	16	E9 (×6)	45,102
					E11 (×4)	
					E13 (×6)	
Kouno *et al.*^53^	THP1-Mac	Temporal gene-expression changes of THP1 single cells differentiating to macrophages	BioMark Dynamic Arrays of Fluidigm for single-cell (Fluidigm Corporation, San Francisco, CA, USA)	240	0 h (×60)	45
					1 h (×60)	
					6 h (×60)	
					96 h (×60)	
Sartor *et al.*^54^	GSE17708	Temporal gene-expression changes during TGF-β-induced epithelial-mesenchymal transition	Affymetrix Human Genome U133 Plus 2.0 Array (Affymetrix Inc.)	15	0 h (×3)	54,675
					8 h (×3)	
					16 h (×3)	
					24 h (×3)	
					72 h (×3)	
