# Supplementary material for: Not just a colourful metaphor: modelling the landscape of cellular development using Hopfield networks
Source: NPJ Syst Biol Appl. 2016 Feb 18;2:16001–. doi: 10.1038/npjsba.2016.1 (PMC5516853; doi:10.1038/npjsba.2016.1)
Supplement: Supplementary Information [file npjsba20161-s1.doc]

**Supplementary information**

**Not just a colourful metaphor: modelling the landscape**

**of cellular development using Hopfield networks**

Atefeh Taherian Fard, Sriganesh Srihari, Jessica C. Mar and Mark A. Ragan

***Generation of random networks:***

For each dataset, we constructed 100 random networks by randomly selecting values from a uniform distribution within the range of the corresponding original gene-expression data. Since all values in each such network are generated randomly, we expect patterns of co-activity between network elements to be non-existent, or extremely rare and without biological meaning. Each set of 100 random networks provides the base against which we compare the original and perturbed states of the corresponding system.

***The three main case-studies:***

The second case study , embryonic mouse brain cells were expression-profiled at embryonic days 9.5, 11.5 and 13.5, the period during which neural progenitor cells progress from proliferation to neuronal differentiation. The dataset encompasses 16 samples: six biological replicates each at 9.5 and 13.5 days, and four at the 11.5-day embryonic stage.

The third case study analyses THP-1 human myeloid monocytic leukaemia cells differentiating to macrophages, monitored at eight time-points post induction with phorbol 12-myristate 13-acetate (PMA). The dataset includes expression profiles from four time-points (0h, 1h, 6h, and 96h) that correspond to the initial, first response, transition and completion stages. In all, 240 single cells (60 single cells per time point) were profiled for 45 TFs. Uniquely among the twelve datasets studied here, in this case study the cells were assayed only for genes already well-known to be involved in the biological process, i.e. the data are not genome-wide.

The fourth case study includes gene-expression profiles from human A549 lung adenocarcinoma cell lines undergoing TGF-beta-induced epithelial-to-mesenchymal transition (EMT), during which cancer cells lose their epithelial characteristics (cell polarity and cell-to-cell adhesion) and become migratory mesenchymal cells. This is an example of transdifferentiation between two stable states. The dataset encompasses 15 samples, three replicates for each of the time-points 0h, 8h, 16h, 24h and 72h post TGF-beta induction.

***Results of the three main case-studies:***

*Case study 2: Development of embryonic mouse brain cells*

The second dataset consisted of 16 samples in three groups assayed with 45,102 expression probes, of which 2748 remained after feature selection. In this case study (GSE8091) we observed a similar energy profile as above. Mouse embryonic brain cells start from a relatively low energy level (E_9.5_ = -1690452) and transition through an intermediate stage (E_11.5_ = -270148) before reaching a differentiated stage with the lowest energy (E_13.5_ = -2024632) (Figures 2 and 4). As before, perturbation analysis showed that the first and last stages are the most resistant to perturbation, whereas the transient stage was more susceptible to perturbation (Figure 3).

The set of the top 100 genes switched between the first and last groups are enriched in annotation for the GO Biological Process terms *Systems development* and *Cell differentiation*, and for the KEGG pathway *Focal adhesion* (Tables 2 and Table S2). Among the enriched genes we find markers of mouse embryonic-cell differentiation including GAP43 and Neurog2 (up-regulated at 13.5 days) and pluripotency markers including Hes5 and Notch1 (up-regulated at 9.5 days) (Figure S1).

*Case study 3: Differentiation of THP1 cells to macrophages*

The third dataset included 240 samples in four groups, assayed for 45 TFs. Due to the small number of probes, we did not carry out feature selection. The initial (0h) and final (96h) stages exhibited the lowest energies, indicating that THP-1 cells (0h) themselves are phenotypically stable. However, upon induction they transit through intermediate-energy stages (1h and 6h) before taking on the behaviour of macrophages (96h) (Figures 2 and 4). Results of perturbation analysis were consistent with this interpretation (Figure 3). Differentiation markers including BCL6 and MAFB were up-regulated, while pluripotency markers such as MYB and RUNX1 were down-regulated at the later stages of development (Figure S1). The set of top switched genes is enriched in annotation for the GO Biological Processes *Cell differentiation* and *System development*, and for the KEGG pathway *MAPK signalling*, which is involved in cell differentiation (Tables 2 and S2). Among the top switched genes we find differentiation markers of THP1 cells BCL6 and MAFB, which were up-regulated at 96h, and the pluripotency markers MYB and RUNX1, which were up-regulated at 0h.

*Case study 4: Cancer cells undergoing EMT*

The fourth dataset consisted of 15 samples, assayed for 54,675 expression probes of which 2620 remained after feature selection. In this case study (GSE17708), human cancer cells exhibit the lowest energies in the two phenotypically stable stages: epithelial (0h, E_0h_ = -401073) and mesenchymal (72h, E_72h_ = -304852). During the intermediate stages of transdifferentiation, the cells pass through stages with higher energies (E_8h_ = -304722, E_16h_ = -146059) (Figures 2 and 4). Perturbation analysis confirms this interpretation, as the initial (0h) and final (72h) stages are more resistant to perturbation than are the intermediate stages (Figure 3).

The GO Biological Process terms most-significantly enriched in annotations of the top switched genes are *Regulation of cell proliferation* and *Developmental process*, and the most-enriched KEGG pathway is *Extracellular matrix-receptor interaction* (Tables 2 and S2). Among the top switched genes are SNAI and ZEB TFs, which are known to play crucial roles in EMT , as well as Sox9 and TGFBR3, which play key roles in development (Figure S1). These enriched functions clearly capture important biology at play the trans-differentiation of immobile epithelial cells to mobile mesenchymal cells.

***Additional eight case-studies:***

We constructed Hopfield network from eight additional time-course datasets (Tables S1, S3 and Figures S2 and S3). *Human hepatocyte differentiation (GSE25417)*

Human pluripotent stem cells were induced to differentiate to hepatocyte-like cells under highly defined culture condition, mimicking the many developmental stages normally associated with the formation of hepatocytes *in vivo*. RNA was isolated from three independent experiments at each stage of differentiation, on days 5, 10, 15 and 20 post-induction .

*Myoblast differentiation (GSE3749)*

This study includes time-course gene expression analysis on differentiating embryonic bodies from a murine J1 embryonic stem cell line over period of two weeks, to identify genes that drive this differentiation process. In this study we used three time-points (the first 12 hours) representing the early stages of differentiation during which the main of loss of pluripotency occurs .

*Human and mouse early embryo development (GSE18290)*

*Human* and *mouse* embryos were harvested at successive stages from oocyte to blastocytes. Time-course gene-expression data were measured at different stages of early development, revealing information on dynamic expression patterns of genes in the whole genomes .

*Human embryo development (GSE18887)*

Three independent human embryos were monitored at six successive developmental stages, Carnegie Stages 9 to 14 (E20 to E32), covering the first third of organogenesis. This study represents the first comprehensive gene expression database of early human organogenesis . *Mouse lung organogenesis (GSE20954)*

Messenger RNA of two mouse embryo (biological replicates) was profiled at all recognized stages of lung development beginning at embryonic day 12 and continuing to adulthood including embryonic days 14, 16 and 18, and postnatal days 2, 10 and 30 .

*Mouse ocular lens development (GSE32334)*

Embryonic lenses from mice were microdissected at embryonic stages E10.5, E11.5 and E12.5 (in three replicates at each time-point) to identify lens-specific gene expression enrichment in these developmental stages .

*Time-course expression profiling of murine cell line transduced with an epitope-tagged form of Hoxa9 (GSE21299)*

Hox genes including Hoxa9 are key regulators of stem cell self-renewal and haematopoiesis. Hoxa9 up-regulation results in early hematopoietic progenitor cells and promotes stem cell expansion; by contrasts its down-regulation is associated with hematopoietic differentiation. Bone-marrow cells were harvested from female 6-8 week old mice and transduced with an MSCV-based retrovirus expressing Hoxa9 fused to a modified estrogen receptor ligand-binding domain (Hoxa9-ER). RNA was extracted at days 0, 3, 4 and 5 (in three replicates at each time-point) for whole-genome gene expression profiling .

***Software and tools***

Implementation and analysis of HNs was performed using open-source libraries in Python 2.7. We used the method and implementation of Maetschke and Ragan to visualize the Hopfield energy landscape. Statistical analysis and visualization were performed using R 3.0.1. Functional and gene-set enrichment analyses were performed using GO and DAVID .


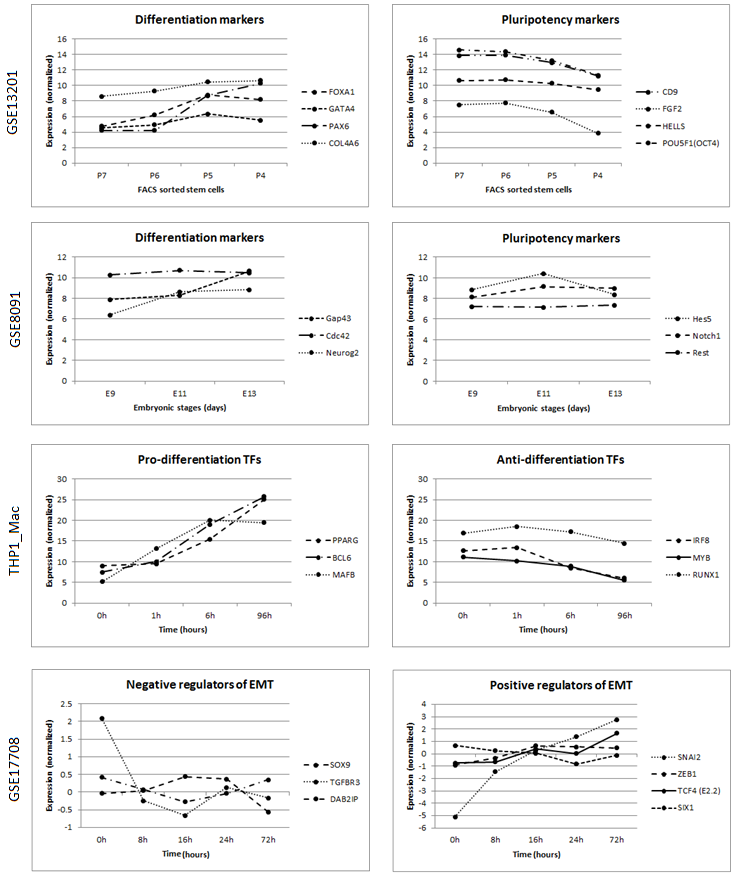


Figure S1: Time-course gene expression of selected TF markers for the four main case-studies.

Table S1: Additional datasets employed in this study.

| Study | Dataset | Summary | Platform | # of samples | Stages of differentiation/development | # of probes |
| --- | --- | --- | --- | --- | --- | --- |
| DeLaForest *et al.*  2011 | GSE25417 | Hepatocyte differentiation  of H9 ES cells (day 0) to definitive endoderm (day 5) and hepatic progenitor cells (day 10) | [HG-U133_Plus_2] Affymetrix Human Genome U133 Plus 2.0 Array | 12 | Day 5 X 3 | 54,675 |
|  |  |  |  |  | Day 10 X 3 |  |
|  |  |  |  |  | Day 15 X 3 |  |
|  |  |  |  |  | Day 20 X 3 |  |
| Hailesellasse Sene *et al.* 2007 | GSE3749 | Mouse embryonic stem cell (mESC) differentiation  11-Point time-course study of differentiating mESC (J1 mESC lines) | [MOE430A] Affymetrix Mouse Expression 430A Array | 9 | 0 h X 3 | 22,690 |
|  |  |  |  |  | 6 h X 3 |  |
|  |  |  |  |  | 12 h X 3 |  |
| Xie *et al.* 2010 | GSE18290  (Mouse) | Early embryo development  Mouse embryo expression data were generated at the one-, two-, four- and eight-cell stages, and at morula, and blastocyst | [MOE430A] Affymetrix Mouse Expression 430A Array | 18 | Stage 1 X 3 | 22,690 |
|  |  |  |  |  | Stage 2 X 3 |  |
|  |  |  |  |  | Stage 4 X 3 |  |
|  |  |  |  |  | Stage 8 X 3 |  |
|  |  |  |  |  | Morula X 3 |  |
|  |  |  |  |  | Blastocyst X 3 |  |
| Xie *et al.* 2010 | GSE18290  (Human) | Early embryo development  Human embryo expression data were generated at the one-, two-, four- and eight-cell stages, and at morula, and blastocyst | [HG-U133_Plus_2] Affymetrix Human Genome U133 Plus 2.0 Array | 18 | Stage 1 X 3 | 54,675 |
|  |  |  |  |  | Stage 2 X 3 |  |
|  |  |  |  |  | Stage 4 X 3 |  |
|  |  |  |  |  | Stage 8 X 3 |  |
|  |  |  |  |  | Morula X 3 |  |
|  |  |  |  |  | Blastocyst X 3 |  |
| Fang *et al.*  2010 | GSE18887 | Early human organogenesis: Transcriptome profiles of human embryos at six successive developmental stages (Carnegie Stages 9 to 14) Human post-implantation embryos were collected at six successive time periods: Carnegie Stages 9 to 14 (E20 to E32) covering the first third of organogenesis | [HG-U133A] Affymetrix Human Genome U133A Array | 18 | E20 X 3 | 22,283 |
|  |  |  |  |  | E22 X 3 |  |
|  |  |  |  |  | E24 X 3 |  |
|  |  |  |  |  | E26 X 3 |  |
|  |  |  |  |  | E28 X 3 |  |
|  |  |  |  |  | E31 X 3 |  |
| Dong *et al.*  2010 | GSE20954 | Mouse lung development  mRNA expression profile in mouse lung development | [Mouse430_2] Affymetrix Mouse Genome 430 2.0 Array | 14 | Day 12 X 2 | 45,101 |
|  |  |  |  |  | Day 14 X 2 |  |
|  |  |  |  |  | Day 16 X 2 |  |
|  |  |  |  |  | Day 18 X 2 |  |
|  |  |  |  |  | Day p-day2 X 2 |  |
|  |  |  |  |  | Day p-day10 X 2 |  |
|  |  |  |  |  | Day p-day30 X 2 |  |
| Lachke *et al.* 2012 | GSE32334 | Embryonic mouse ocular lens development | [Mouse430_2] Affymetrix Mouse Genome 430 2.0 Array | 9 | E10.5 X 3 | 45,101 |
|  |  |  |  |  | E11.5 X 3 |  |
|  |  |  |  |  | E12.5 X 3 |  |
| Muntean *el al.* 2012 | GSE21299 | Bone marrow cells collected from 6-8 week female mice were transduced with a Hoxa9 expressing retrovirus. Hoxa9-ER cells were sampled at different intervals | [Mouse430_2] Affymetrix Mouse Genome 430 2.0 Array | 12 | Day 0 X 3 | 45,101 |
|  |  |  |  |  | Day 3 X 3 |  |
|  |  |  |  |  | Day 4 X 3 |  |
|  |  |  |  |  | Day 5 X 3 |  |

Table S3: Overview of case studies. For each case study, the proportion of variance captured by principal components PC1 and PC2; energy scores for each time-point or group; number of feature-selected genes or probes; and number of probes or genes observed to switch expression at each transition.

| Case study | PC1(%) | PC2(%) | Time-point (groups) | *E* | # selected probes | Transition | # probes switched |
| --- | --- | --- | --- | --- | --- | --- | --- |
| GSE13201 | 52.01 | 9.24 | P7 | -1320897 | 3,753 | P7 to P4 | 3,405 |
|  |  |  | P6 | -755220 |  | P5 to P4 | 1,861 |
|  |  |  | P5 | -599724 |  | P6 to P4 | 3,430 |
|  |  |  | P4 | -3307223 |  | P6 to P5 | 2,712 |
|  |  |  |  |  |  | P7 to P5 | 2,810 |
|  |  |  |  |  |  | P7 to P6 | 1,453 |
| GSE8091 | 71.56 | 22.21 | E9 | -1690452 | 2,748 | E9 to E13 | 2,594 |
|  |  |  | E11 | -270148 |  | E9 to E11 | 2,169 |
|  |  |  | E13 | -2024632 |  | E11 to E13 | 2,430 |
| THP1-Mac | 17.84 | 10.25 | 0h | -23.9821 | 45 | 0h to 96h | 20 |
|  |  |  | 1h | -10.0384 |  | 0h to 1h | 23 |
|  |  |  | 6h | -7.54461 |  | 0h to 6h | 22 |
|  |  |  | 96h | -22.1606 |  | 1h to 6h | 23 |
|  |  |  |  |  |  | 1h to 96h | 21 |
|  |  |  |  |  |  | 1h to 6h | 23 |
|  |  |  |  |  |  | 6h to 96h | 22 |
| GSE17708 | 29.28 | 11.57 | 0h | -401074 | 2,620 | 0h to 72h | 1,974 |
|  |  |  | 8h | -304722 |  | 0h to 8h | 1,511 |
|  |  |  | 16h | -146059 |  | 0h to 16h | 2,013 |
|  |  |  | 24h | -273684 |  | 0h to 24h | 2,021 |
|  |  |  | 72h | -304852 |  | 8h to 16h | 1,806 |
|  |  |  |  |  |  | 16h to 24h | 1,606 |
|  |  |  |  |  |  | 16h to 72h | 1,737 |
|  |  |  |  |  |  | 8h to 24h | 1,936 |
|  |  |  |  |  |  | 24h to72h | 1,478 |
|  |  |  |  |  |  | 8h to 72h | 1,960 |
| GSE25417 | 43.32 | 19.13 | Day 5 | -2569512 | 5,042 | Day 5 to Day 20 | 4000 |
|  |  |  | Day 10 | -1742933 |  |  |  |
|  |  |  | Day 15 | -1542097 |  |  |  |
|  |  |  | Day 20 | -2720105 |  |  |  |
| GSE3749 | 15.95 | 13.46 | 0 h | -201347 | 2,368 | 0h to 12 h | 1704 |
|  |  |  | 6 h | -190764 |  |  |  |
|  |  |  | 12 h | -199198 |  |  |  |
| GSE18290  (Mouse) | 53.9 | 25.64 | Stage 1 | -440921 | 1,827 | Stage1 to Blastocyst | 1,488 |
|  |  |  | Stage 2 | -603086 |  |  |  |
|  |  |  | Stage 4 | -416632 |  |  |  |
|  |  |  | Stage 8 | -385209 |  |  |  |
|  |  |  | Morula | -579501 |  |  |  |
|  |  |  | Blastocyst | -389707 |  |  |  |
| GSE18290  (Human) | 73.91 | 8.5 | Stage 1 | -6414379 | 4,684 | Stage1 to Blastocyst | 4,478 |
|  |  |  | Stage 2 | -6016848 |  |  |  |
|  |  |  | Stage 4 | -5081828 |  |  |  |
|  |  |  | Stage 8 | -4612578 |  |  |  |
|  |  |  | Morula | -6507029 |  |  |  |
|  |  |  | Blastocyst | -6067749 |  |  |  |
| GSE18887 | 58.69 | 15.79 | E20 | -222282 | 1,050 | E20 to E31 | 927 |
|  |  |  | E22 | -218827 |  |  |  |
|  |  |  | E24 | -111771 |  |  |  |
|  |  |  | E26 | -101452 |  |  |  |
|  |  |  | E28 | -226820 |  |  |  |
|  |  |  | E31 | -231239 |  |  |  |
| GSE20954 | 79.61 | 8.66 | Day 12 | -936343 | 3,799 | Day 12 to Day p-Day 30 | 2,868 |
|  |  |  | Day 14 | -675155 |  |  |  |
|  |  |  | Day 16 | -172117 |  |  |  |
|  |  |  | Day 18 | -541078 |  |  |  |
|  |  |  | Day p-day2 | -551912 |  |  |  |
|  |  |  | Day p-day10 | -501771 |  |  |  |
|  |  |  | Day p-day30 | -1508509 |  |  |  |
| GSE32334 | 56.38 | 22.56 | E10.5 | -743351 | 2,060 | E10.5 to E12.5 | 1,602 |
|  |  |  | E11.5 | -341230 |  |  |  |
|  |  |  | E12.5 | -722994 |  |  |  |
| GSE21299 | 47.72 | 19.67 | Day 0 | -1034284 | 2,804 | Day 0 to Day 5 | 2,804 |
|  |  |  | Day 3 | -554739 |  |  |  |
|  |  |  | Day 4 | -511360 |  |  |  |
|  |  |  | Day 5 | -1152137 |  |  |  |


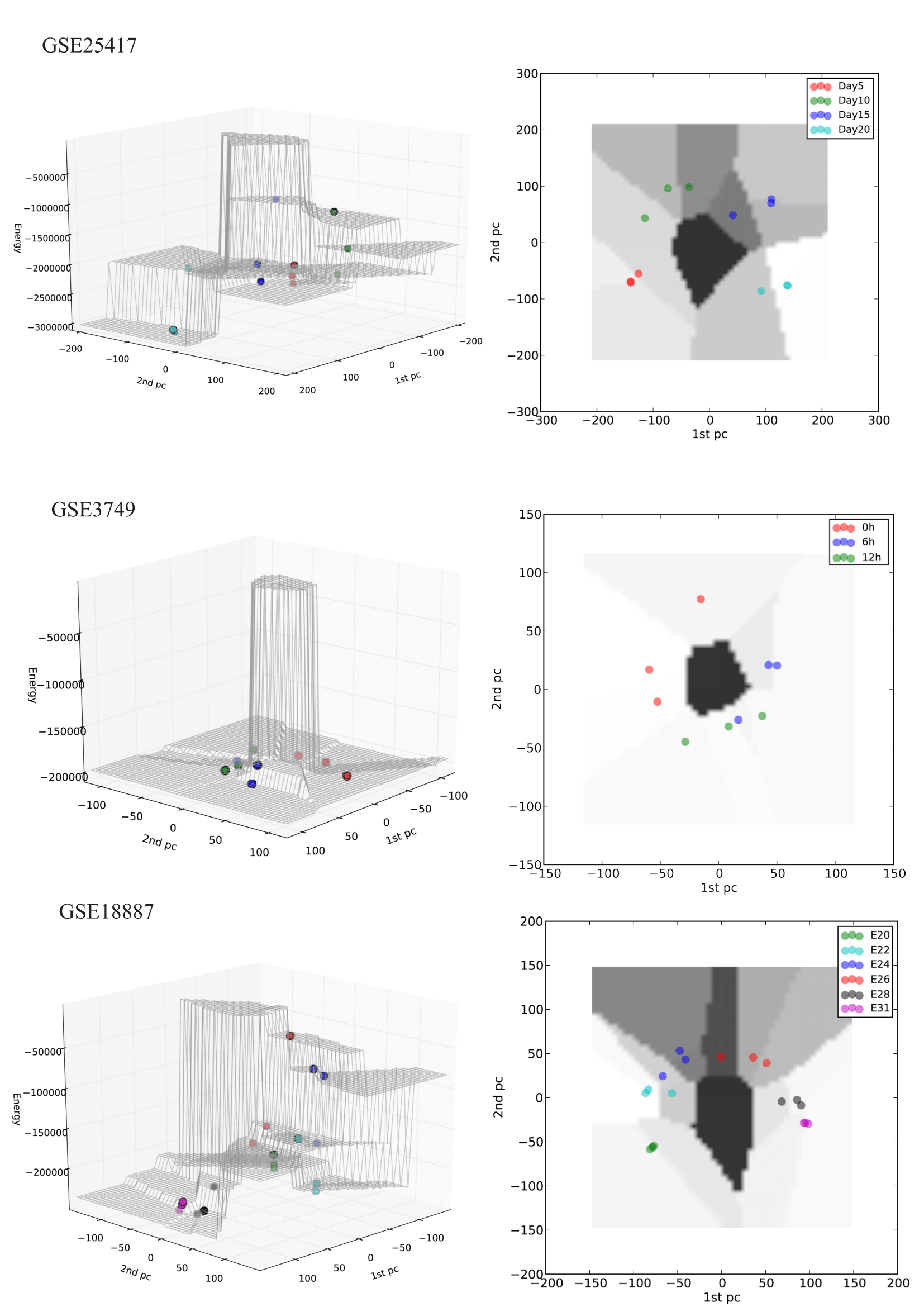


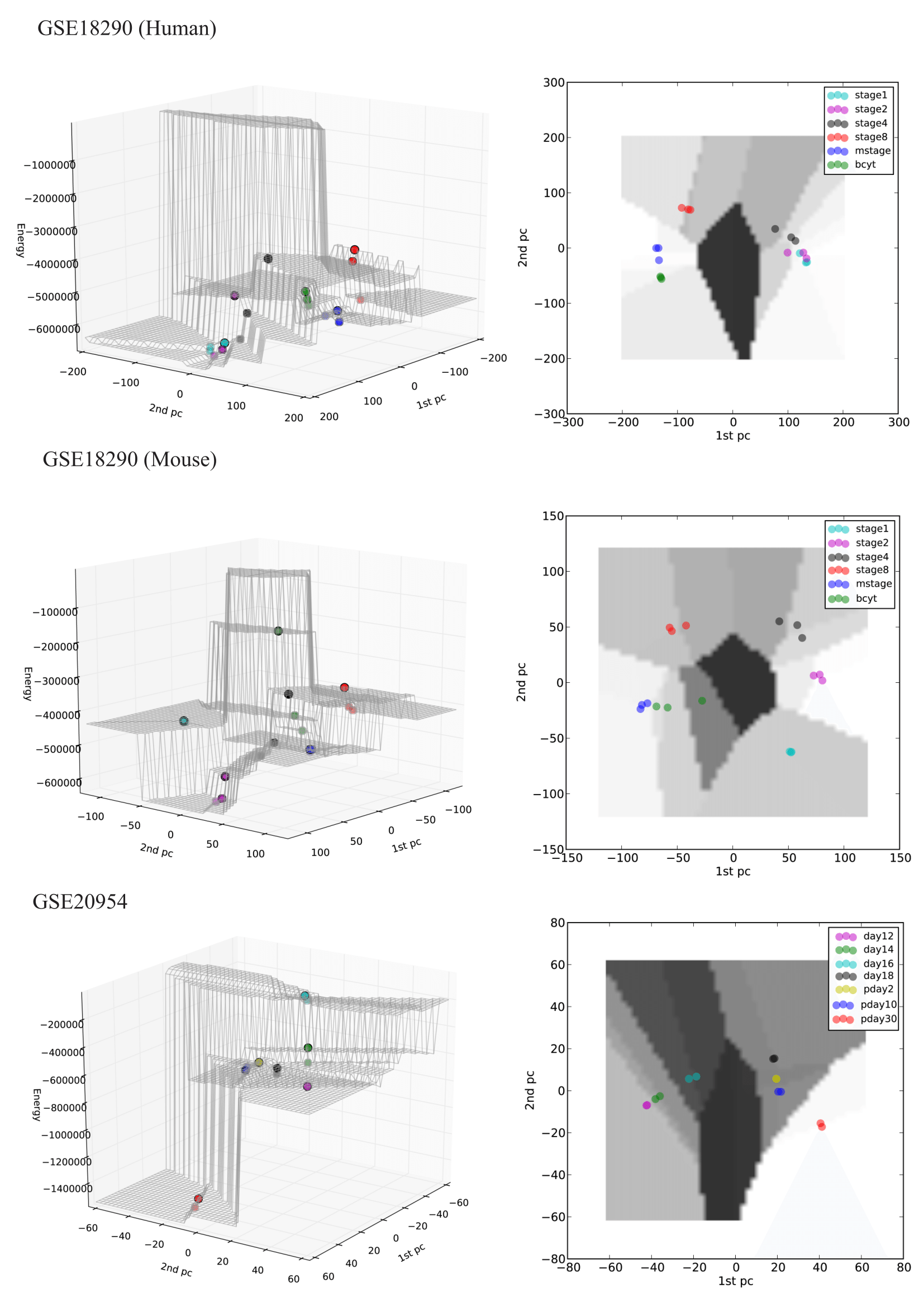


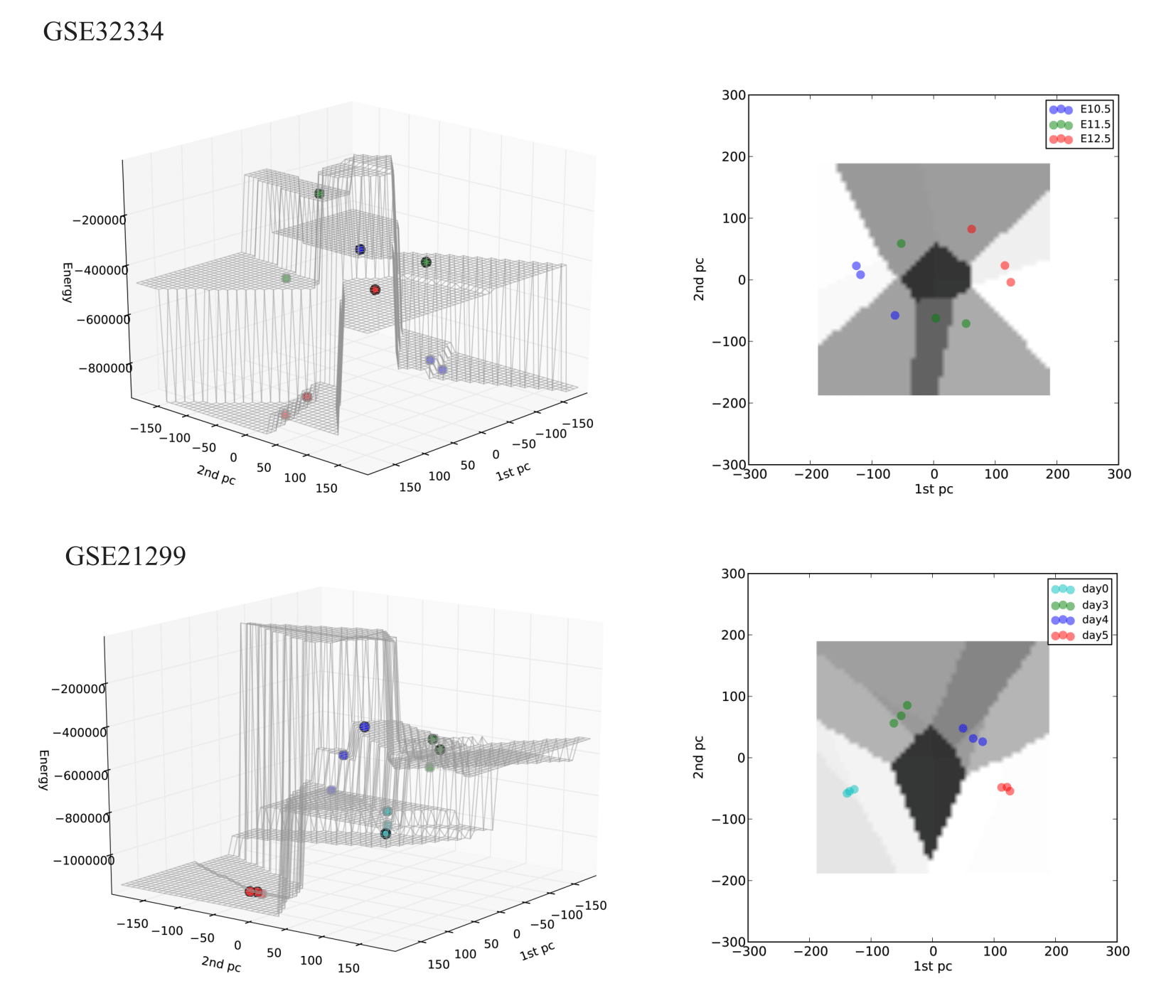


Figure S2: Hopfield energy landscape of the eight additional datasets.

GSE18290 (Human)

GSE18887


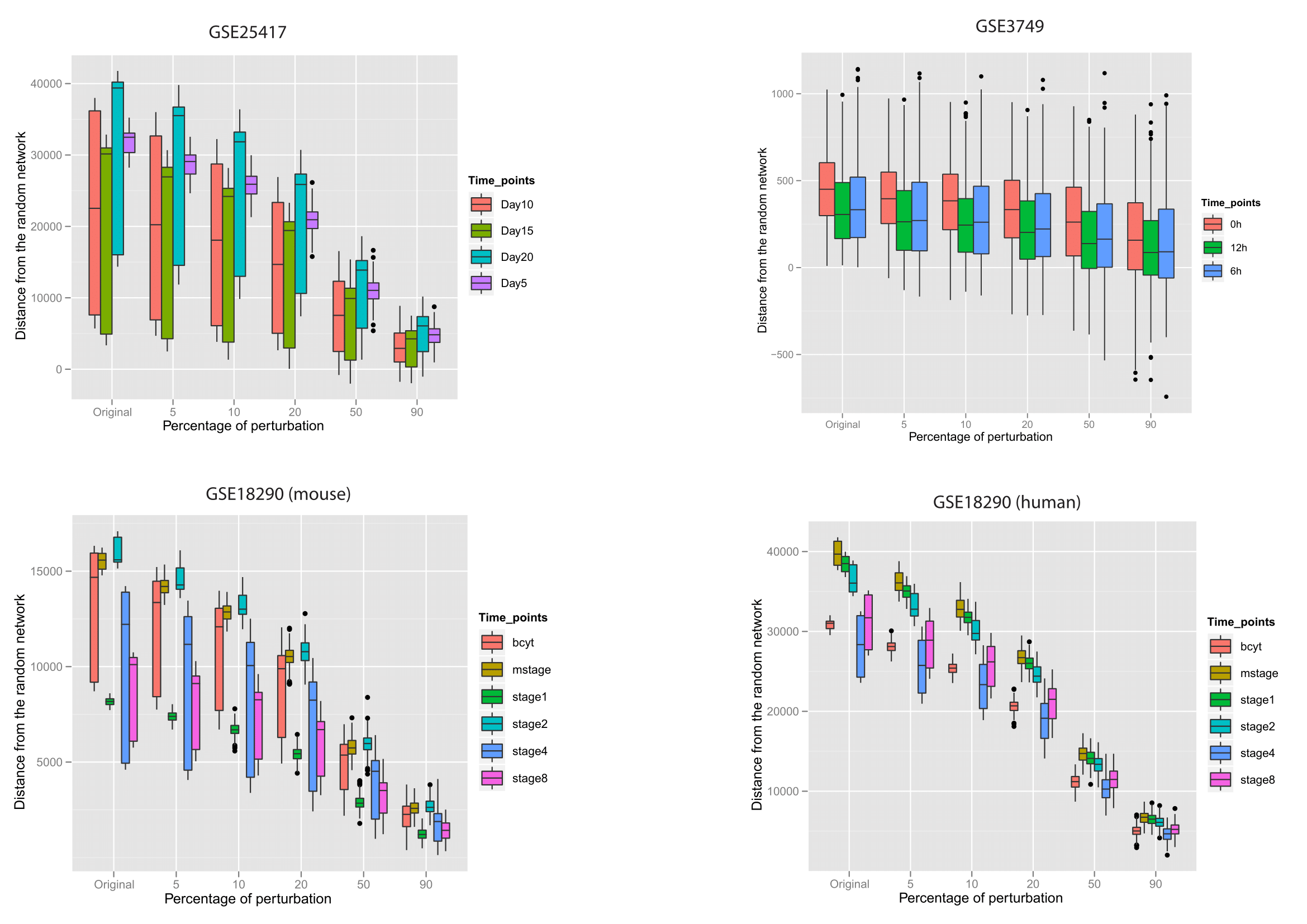


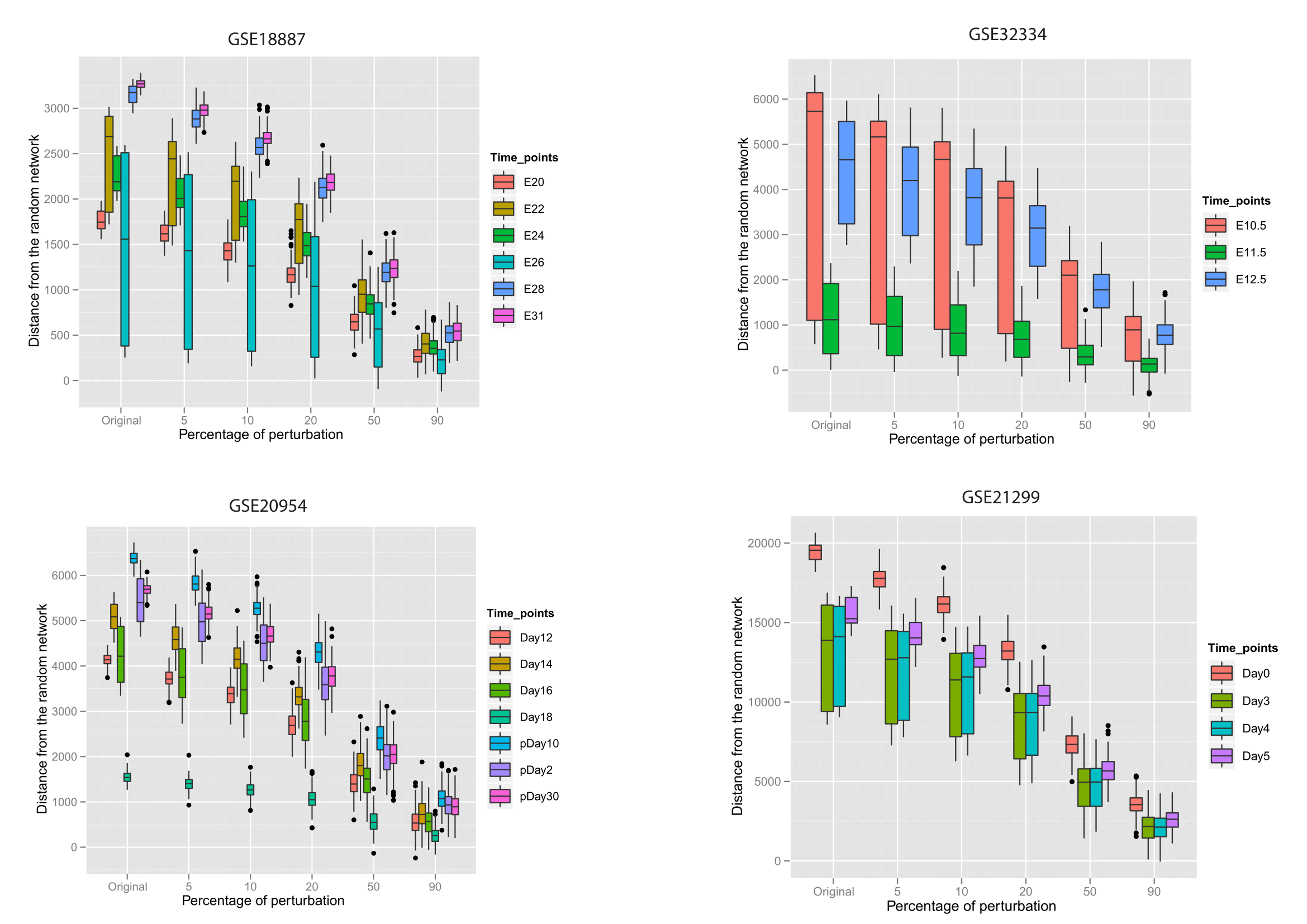


Figure S3: Differences between the means of energies for cellular stages in the additional eight case studies.

**References**

1. Hartl D, Irmler M, Römer I, Mader MT, Mao L, Zabel C, et al. Transcriptome and proteome analysis of early embryonic mouse brain development. *Proteomics* 2008;**8(6)**:1257-65.

2. Kouno T, de Hoon M, Mar J, Tomaru Y, Kawano M, Carninci P, *et al*. Temporal dynamics and transcriptional control using single-cell gene expression analysis. *Genome Biology* 2013;**14(10)**:R118.

3. Sartor MA, Mahavisno V, Keshamouni VG, Cavalcoli J, Wright Z, Karnovsky A, *et al*. ConceptGen: a gene set enrichment and gene set relation mapping tool. *Bioinformatics* 2010;**26(4)**:456-63.

4. Takeda K, Ichijo H. Neuronal p38 MAPK signalling: an emerging regulator of cell fate and function in the nervous system. *Genes to Cells* 2002;**7(11)**:1099-111.

5. Danen EHJ, Yamada KM. Fibronectin, integrins, and growth control. *Journal of Cellular Physiology* 2001;**189(1)**:1-13.

6. Mitra SK, Hanson DA, Schlaepfer DD. Focal adhesion kinase: in command and control of cell motility. *Nat Rev Mol Cell Biol* 2005;**6(1)**:56-68.

7. Peinado H, Olmeda D, Cano A. Snail, Zeb and bHLH factors in tumour progression: an alliance against the epithelial phenotype? *Nat Rev Cancer*. 2007;**7(6)**:415-28.

8. Weimin Bi JMD, Zhaoping Zhang, Richard R. Behringer, and Benoit de Crombrugghe. Sox9 is required for cartilage formation. *Nature Genetics* 1999;**22**:4.

9. Lim J, Thiery JP. Epithelial-mesenchymal transitions: insights from development. *Development* 2012;**139(19)**:3471-86.

10. DeLaForest A, Nagaoka M, Si-Tayeb K, Noto FK, Konopka G, Battle MA, *et al*. HNF4A is essential for specification of hepatic progenitors from human pluripotent stem cells. *Development* (Cambridge, England) 2011;**138(19)**:4143-53.

11. Sene KH, Porter CJ, Palidwor G, Perez-Iratxeta C, Muro EM, Campbell PA, *et al*. Gene function in early mouse embryonic stem cell differentiation. *BMC Genomics* 2007;**8**:85-.

12. Xie D, Chen C-C, Ptaszek LM, Xiao S, Cao X, Fang F, *et al*. Rewirable gene regulatory networks in the preimplantation embryonic development of three mammalian species. *Genome Research* 2010;**20(6)**:804-15.

13. Fang H, Yang Y, Li C, Fu S, Yang Z, Jin G, *et al*. Transcriptome Analysis of Early Organogenesis in Human Embryos. *Developmental Cell* 2010;**19(1)**:174-84.

14. Dong J, Jiang G, Asmann YW, Tomaszek S, Jen J, Kislinger T, *et al*. MicroRNA Networks in Mouse Lung Organogenesis. *PLoS ONE* 2010;**5(5)**:e10854.

15. Lachke SA, Ho JWK, Kryukov GV, O'Connell DJ, Aboukhalil A, Bulyk ML, *et al*. iSyTE: Integrated Systems Tool for Eye Gene Discovery. *Investigative Ophthalmology & Visual Science* 2012;**53(3)**:1617-27.

16. Muntean AG, Tan J, Sitwala K, Huang Y, Bronstein J, Connelly JA, *et al*. The PAF complex synergizes with MLL fusion proteins at HOX loci to promote leukemogenesis. *Cancer Cell* 2010;**17(6)**:609-21.

17. Maetschke SR, Ragan MA. Characterizing cancer subtypes as attractors of Hopfield networks. *Bioinformatics* 2014;**30(9)**:1273-9.

18. Ashburner M, Ball CA, Blake JA, Botstein D, Butler H, Cherry JM, et al. Gene Ontology: tool for the unification of biology. *Nat Genet* 2000;**25(1)**:25-9.

19. Huang DW, Sherman BT, Lempicki RA. Systematic and integrative analysis of large gene lists using DAVID bioinformatics resources. *Nat Protocols* 2008;**4(1)**:44-57.

20. Huang DW, Sherman BT, Lempicki RA. Bioinformatics enrichment tools: paths toward the comprehensive functional analysis of large gene lists. *Nucleic Acids Research* 2009;**37(1)**:1-13.
