# Supplementary material for: Not just a colourful metaphor: modelling the landscape of cellular development using Hopfield networks
Source: NPJ Syst Biol Appl. 2016 Feb 18;2:16001–. doi: 10.1038/npjsba.2016.1 (PMC5516853; doi:10.1038/npjsba.2016.1)
Supplement: Supplementary Table S1 [file npjsba20161-s2.pdf]

Table S1: Additional datasets employed in this study.

Study	Dataset	Summary	Platform	# of samples	Stages of differentiation/development	# of probes
DeLaForest *et al.* 2011	GSE25417	Hepatocyte differentiation of H9 ES cells (day 0) to definitive endoderm (day 5) and hepatic progenitor cells (day 10)	[HG-U133_Plus_2] Affymetrix Human Genome U133 Plus 2.0 Array	12	Day 5 X 3	54,675
					Day 10 X 3	
					Day 15 X 3	
					Day 20 X 3	
Hailesellasse Sene *et al.* 2007	GSE3749	Mouse embryonic stem cell (mESC) differentiation 11-Point time-course study of differentiating mESC (J1 mESC lines)	[MOE430A] Affymetrix Mouse Expression 430A Array	9	0 h X 3	22,690
					6 h X 3	
					12 h X 3	
Xie *et al.* 2010	GSE18290 (Mouse)	Early embryo development Mouse embryo expression data were generated at the one-, two-, four- and eight-cell stages, and at morula, and blastocyst	[MOE430A] Affymetrix Mouse Expression 430A Array	18	Stage 1 X 3	22,690
					Stage 2 X 3	
					Stage 4 X 3	
					Stage 8 X 3	
					Morula X 3	
					Blastocyst X 3	
Xie *et al.* 2010	GSE18290 (Human)	Early embryo development Human embryo expression data were generated at the one-, two-, four- and eight-cell stages, and at morula, and blastocyst	[HG-U133_Plus_2] Affymetrix Human Genome U133 Plus 2.0 Array	18	Stage 1 X 3	54,675
					Stage 2 X 3	
					Stage 4 X 3	
					Stage 8 X 3	
					Morula X 3	
					Blastocyst X 3	
Fang *et al.* 2010	GSE18887	Early human organogenesis: Transcriptome profiles of human embryos at six successive developmental stages (Carnegie Stages 9 to 14) Human post-implantation embryos were collected at six successive time periods: Carnegie Stages 9 to 14 (E20 to E32) covering the first third of organogenesis	[HG-U133A] Affymetrix Human Genome U133A Array	18	E20 X 3	22,283
					E22 X 3	
					E24 X 3	
					E26 X 3	
					E28 X 3	

					E31 X 3	
Dong *et al.* 2010	GSE20954	Mouse lung development<br>mRNA expression profile in mouse lung development	[Mouse430_2] Affymetrix Mouse Genome 430 2.0 Array	14	Day 12 X 2	45,101
					Day 14 X 2	
					Day 16 X 2	
					Day 18 X 2	
					Day p-day2 X 2	
					Day p-day10 X 2	
					Day p-day30 X 2	
Lachke *et al.* 2012	GSE32334	Embryonic mouse ocular lens development	[Mouse430_2] Affymetrix Mouse Genome 430 2.0 Array	9	E10.5 X 3	45,101
					E11.5 X 3	
					E12.5 X 3	
Muntean *el al.* 2012	GSE21299	Bone marrow cells collected from 6-8 week female mice were transduced with a Hoxa9 expressing retrovirus. Hoxa9-ER cells were sampled at different intervals	[Mouse430_2] Affymetrix Mouse Genome 430 2.0 Array	12	Day 0 X 3	45,101
					Day 3 X 3	
					Day 4 X 3	
					Day 5 X 3	
