# Supplementary material for: Not just a colourful metaphor: modelling the landscape of cellular development using Hopfield networks
Source: NPJ Syst Biol Appl. 2016 Feb 18;2:16001–. doi: 10.1038/npjsba.2016.1 (PMC5516853; doi:10.1038/npjsba.2016.1)
Supplement: Supplementary Table S3 [file npjsba20161-s4.pdf]

Table S3: Overview of case studies. For each case study, the proportion of variance captured by principal components PC1 and PC2; energy scores for each time-point or group; number of feature-selected genes or probes; and number of probes or genes observed to switch expression at each transition.

Case study	PC1(%)	PC2(%)	Time-point (groups)	*E*	# selected probes	Transition	# probes switched
GSE13201	52.01	9.24	P7	-1320897	3,753	P7 to P4	3,405
			P6	-755220		P5 to P4	1,861
			P5	-599724		P6 to P4	3,430
			P4	-3307223		P6 to P5	2,712
						P7 to P5	2,810
						P7 to P6	1,453
GSE8091	71.56	22.21	E9	-1690452	2,748	E9 to E13	2,594
			E11	-270148		E9 to E11	2,169
			E13	-2024632		E11 to E13	2,430
THP1-Mac	17.84	10.25	0h	-23.9821	45	0h to 96h	20
			1h	-10.0384		0h to 1h	23
			6h	-7.54461		0h to 6h	22
			96h	-22.1606		1h to 6h	23
						1h to 96h	21
						1h to 6h	23
						6h to 96h	22
GSE17708	29.28	11.57	0h	-401074	2,620	0h to 72h	1,974
			8h	-304722		0h to 8h	1,511
			16h	-146059		0h to 16h	2,013
			24h	-273684		0h to 24h	2,021
			72h	-304852		8h to 16h	1,806
						16h to 24h	1,606
						16h to 72h	1,737
						8h to 24h	1,936
						24h to72h	1,478
						8h to 72h	1,960
GSE25417	43.32	19.13	Day 5	-2569512	5,042	Day 5 to Day 20	4000
			Day 10	-1742933			
			Day 15	-1542097			
			Day 20	-2720105			
GSE3749	15.95	13.46	0 h	-201347	2,368	0h to 12 h	1704
			6 h	-190764			
			12 h	-199198			
GSE18290 (Mouse)	53.9	25.64	Stage 1	-440921	1,827	Stage1 to Blastocyst	1,488
			Stage 2	-603086			
			Stage 4	-416632			
			Stage 8	-385209			
			Morula	-579501			
			Blastocyst	-389707			
GSE18290	73.91	8.5	Stage 1	-6414379	4,684		4,478

(Human)			Stage 2	-6016848		Stage1 to Blastocyst	
			Stage 4	-5081828			
			Stage 8	-4612578			
			Morula	-6507029			
			Blastocyst	-6067749			
GSE18887	58.69	15.79	E20	-222282	1,050	E20 to E31	927
			E22	-218827			
			E24	-111771			
			E26	-101452			
			E28	-226820			
			E31	-231239			
GSE20954	79.61	8.66	Day 12	-936343	3,799	Day 12 to Day p-Day 30	2,868
			Day 14	-675155			
			Day 16	-172117			
			Day 18	-541078			
			Day p-day2	-551912			
			Day p-day10	-501771			
			Day p-day30	-1508509			
GSE32334	56.38	22.56	E10.5	-743351	2,060	E10.5 to E12.5	1,602
			E11.5	-341230			
			E12.5	-722994			
GSE21299	47.72	19.67	Day 0	-1034284	2,804	Day 0 to Day 5	2,804
			Day 3	-554739			
			Day 4	-511360			
			Day 5	-1152137			
